# Impact of Lead Pollution from Vehicular Traffic on Highway-Side Grazing Areas: Challenges and Mitigation Policies

**DOI:** 10.3390/ijerph22020311

**Published:** 2025-02-19

**Authors:** Tareq A. Al-Sabbagh, Sheikh Shreaz

**Affiliations:** Food Security Program, Environment and Life Sciences Research Center, Kuwait Institute for Scientific Research, P.O. Box 24885, Safat 13109, Kuwait

**Keywords:** lead contamination, highways, sheep grazing, public health, livestock management, soil remediation

## Abstract

One major environmental concern is the lead (Pb) pollution from automobile traffic, especially in highway-side grazing areas. Sheep grazing in Pb-contaminated areas are particularly vulnerable because Pb exposure from soil, water, and feed can have harmful effects that impair their general health, reproductive capability, and immune systems. Long-term hazards to cattle from persistent Pb exposure include neurotoxicity, hematological abnormalities, reproductive health problems, and immunosuppression. These can have serious consequences, such as reduced productivity and even mortality. Additionally, through the food chain, Pb bioaccumulation in lamb tissues directly endangers human health. Pb poisoning is caused by a variety of intricate mechanisms, including disturbances in calcium-dependent processes, oxidative stress, and enzyme inhibition. To mitigate these risks, an interdisciplinary approach is essential, combining expertise in environmental science, toxicology, animal husbandry, and public health. Effective strategies include rotational grazing, alternative foraging options, mineral supplementation, and soil remediation techniques like phytoremediation. Additionally, the implementation of stringent regulatory measures, continuous monitoring, and community-based initiatives are vital. This review emphasizes the need for comprehensive and multidisciplinary methodologies to address the ecological, agricultural, and public health impacts of Pb pollution. By integrating scientific expertise and policy measures, it is possible to ensure the long-term sustainability of grazing systems, protect livestock and human health, and maintain ecosystem integrity.

## 1. Introduction

Animals grazing mostly near highways consume lead (Pb)-contaminated vegetation and soil, posing health risks to animals and humans as their consumers [1]. Leaded gasoline has historically been a major source of Pb pollution in the environment [2,3,4]. Although it has been phased out in many countries, its emissions have left long-lasting deposits in soil. Sheep that graze close to busy roads are therefore especially susceptible to high Pb concentrations.

Heavy metal pollution, including Pb contamination is significantly influenced by other anthropogenic sources, including mining operations, waste disposal, industrial emissions, and submarine discharges [5,6,7]. Almost 19 countries are severely impacted by Pb contamination near lead mining areas, lead smelting areas and due to vehicular emissions of Pb, as shown in Table 1. Pb-exposure routes in these countries differ slightly but most of the countries have similar Pb-exposure sources and routes. Severe cases of Pb poisoning have been reported in Nigeria, which have affected 40% of children, causing nearly 400 deaths [8]. It has been reported that these children were exposed to Pb while coming into contact with Pb-based paints and batteries. Around the world, in various places like Norway and Switzerland, sheep are exposed to Pb in unfenced shooting range areas near animal grazing fields [9]. A study showed that not only were high Pb contents found in these animals but the maximum amounts of copper were also present in blood, causing severe changes in physiology and increased death rates.

Ingested Pb can interfere with enzymatic processes, cause neurological disorders, lead to anemia, and reduce reproductive capabilities [10]. It has also been reported that chronic lead exposure at low levels negatively impacts growth rates and the quality of meat [11]. Pb content is found in the muscles and livers of sheep grazing near highways contaminated with Pb. Many studies have reported that the levels of Pb in various organs in lead-exposed sheep have crossed reference ranges [12].

Effective management strategies are necessary to mitigate the risks of Pb contamination; these mitigation strategies include monitoring food and water sources for Pb contamination [13] along selected grazing areas near highways and industrial zones [14]. Some other strategies include nutritional interventions, like incorporating chelating agents and ensuring adequate dietary calcium and phosphorus, which can reduce the absorption of Pb in lambs [15,16].

In this review, we have critically examined the various sources of Pb contamination on the sides of highways; we have also reported how pollutants affect sheep production in contaminated grazing areas. This review aims to identify the sources of Pb pollution near roads and the routes via which it reaches grazing areas and report the effects of Pb exposure on lamb productivity and health. We also discuss approaches to mitigation to minimize Pb ingestion and its impact on consumers. The factors influencing Pb distribution, including the density of traffic along roads with grazing areas, industrial activities, and soil and vegetation characteristics, are explored to provide insights into Pb contamination patterns.

**Table 1:** List of countries with different sources of lead contamination and its impact.

Country	Sources of Lead Contamination	Impact	Most Affected Area	References
Nigeria	Artisanal mining	40% of children affected, over 400 deaths (2010)	Zamfara State (Bagega village)	[8]
China	Industrial emissions, smelting, leaded gasoline	Up to 30% of children in industrial areas with elevated lead levels	Hunan, Henan	[17]
Sweden	Industrial waste management, leaded gasoline	Elevated lead in water and soil near industrial areas	Ronnskar	[17]
France	Industrial activities, smelting, waste disposal	Elevated lead levels in urban soils near industrial sites	Northern France	[18]
Germany	Vehicular emissions, industrial activities	High lead concentrations in urban areas with heavy traffic	Ruhr area	[19]
United States	Water pipes, industrial emissions, lead-based paints	Flint water crisis affected over 100,000 people	Flint, Michigan	[20]
India	Battery manufacturing, e-waste recycling, leaded gasoline	25% of children in industrial areas with elevated lead levels	Delhi, Kolkata	[21,22]
Mexico	Industrial, artisanal sources (pottery with lead glazes)	33% of children with elevated blood lead levels in rural areas	Rural areas (pottery production)	[23]
Peru	Lead mining and smelting	97% of children in La Oroya with elevated blood lead levels	La Oroya	[24,25]
Australia	Mining, smelting	Port Pirie children frequently exceed blood lead limits	Port Pirie	[26]
Brazil	Mining, industrial processes	Elevated lead levels in urban soil and water systems	Santo Amaro da Purificacao	[27]
Iran	Industrial emissions, smelting	Elevated lead levels in industrial cities	Isfahan	[28]
Saudi Arabia	Vehicular emissions, industrial processes, plumbing systems	Rising lead levels in urban areas	Riyadh	[29]
Jordan	Mining activities, lead smelting	Elevated lead concentrations in soil and water	Zarqa	[30]
Iraq	Industrial emissions, oil industry pollution, leaded gasoline	Lead contamination near industrial facilities	Basra, Baghdad	[31]
Lebanon	Urban and industrial pollution, lead pipes	High lead levels in urban soil and water	Beirut	[32]
Oman	Industrial emissions, metal industries	Rising lead contamination in industrial zones	Sohar	[33]
UAE	Vehicular emissions, industrial processes	Lead contamination from past use of leaded gasoline, industrial	Dubai, Abu Dhabi	[34]

## 2. Sources and Pathways of Lead Contamination

Pb contamination can arise from both natural and anthropogenic sources. Natural sources include lead sulfide (PbS) deposits in the earth’s crust and the weathering processes of rocks. Conversely, anthropogenic sources are human made, encompassing mining activities (e.g., Pb poisoning incidents in Zamfara State, Nigeria), vehicle emissions (notably from leaded gasoline), smelting industries, electrical waste dismantling, and various manufacturing processes, including paint and glass production, agricultural practices, waste disposal, and plumbing fixtures containing Pb. Table 1 mentions some countries with different sources of Pb contamination and its impact on human health [5].

### 2.1. Vehicular Emissions

Pb is a universal environmental pollutant that poses significant risks to both ecosystems and human health. It enters the environment through various sources, including industrial, domestic, and commercial activities. Major contributors to Pb contamination include leaded gasoline, diesel exhaust, mining, smelting, battery manufacturing, paint production, improper disposal of electronic waste, and waste incineration [35]. Historically, automobile emissions were a major source of Pb deposition, primarily due to the widespread use of leaded gasoline [36].

Leaded gasoline, in the form of tetraethyl lead (TEL), was once extensively used to enhance engine performance by preventing knocking and was a significant source of environmental soil pollution in the 20th century [37]. However, as evidence of the health risks associated with Pb exposure mounted, many countries began phasing out leaded gasoline in the latter half of the 20th century [38]. Studies from that period revealed elevated blood lead levels in children living near heavily trafficked areas [39]. Pb particles released from car engines during combustion were deposited on roadsides in soil, water, and vegetation [36]. Due to the recognized health dangers, the global phase-out of leaded gasoline began in the 1970s [40].

Diesel exhaust has also contributed to airborne lead emissions. Pb and other heavy metals are released through diesel combustion and lubricating oil additives [41]. Although regulations on low-sulfur diesel and the use of catalytic converters have significantly reduced heavy metal emissions [42], diesel exhaust remains a source of Pb. Studies have found that both the particulate matter in diesel exhaust and its associated organic components contain trace amounts of environmental contaminants, including Pb [43,44]. While the cessation of leaded gasoline use has drastically reduced airborne lead emissions [19], diesel exhaust continues to contribute to the problem. In Table 2 we have provided some details of Pb contamination through various sources like mining and smelting, lead–acid batteries, paint production, and waste incinerations.

#### Factors Influencing Lead Dispersion and Accumulation near Highways

The dispersion and accumulation of Pb near highways are driven by multiple environmental pathways and influenced by various factors. Wind transports Pb-contaminated dust over long distances before depositing it on the surface of soil, water, or vegetation [48]. Pb particles settle directly on plant leaves or plant roots, can absorb Pb through soil, and may be ingested by animals [49]. These processes create multiple exposure routes for humans, including inhalation, ingestion, and dermal contact [35]. The severity of Pb dispersion near highways depends on factors such as traffic density, vehicle type, emission control technologies, meteorological conditions, and land use. Amato et al. reported that the most important determinant of Pb dispersion near highways is high traffic density [50]. Due to higher traffic density, emissions of Pb from vehicles into the atmosphere increase, which in turn results in greater dispersion and accumulation of Pb [51]. It has also been reported that older vehicles which are using leaded gasoline are responsible for emitting higher levels of Pb [52]. Vehicle type also plays a critical role in Pb dispersion. Older vehicles or those with poor maintenance tend to emit higher levels of Pb [19,52].

Emission control technologies are instrumental in bringing down Pb emissions. These emission control technologies are catalytic converters and exhaust after treatment systems, which have been important in diminishing Pb emissions from vehicles over recent decades [53,54,55]. However, these technologies are not always 100% effective in eliminating Pb particle emissions. Meteorological conditions also play a crucial role in determining the dispersion of Pb particles. Factors such as wind direction, wind speed, temperature, and humidity can affect the range and distance Pb particles travel before settling [56].

Pb released by vehicles near highways has a great effect on patterns of Pb deposition. The presence of areas of vegetation and green spaces alongside roads can act as barriers that filter airborne pollutants, such as Pb, reducing its deposition on soil and water surfaces [57]. Trees or other vegetation help to decrease the spread of Pb, while Pb particles are likely to accumulate over impervious surfaces in commercial or industrial land use. Here, Pb may be washed off by flood and stormwater runoff and lead to the contamination of adjacent water bodies like lakes and streams. Other activities like smelting and mining also play a major role in Pb contamination according to researchers [58]. Fine particulate matter containing Pb can be dispersed by the wind and deposited on soils far from its source.

Contaminated surface soils can act as secondary sources of Pb pollution, affecting nearby communities. Exposure to Pb contamination may occur through direct contact with the soil, inhalation of suspended particles, or consumption of plants and water from contaminated areas [59]. Table 3 provides concise information on the factors influencing Pb dispersion.

### 2.2. Impact of Industrial Activities and Contamination Routes on Lead Pollution in Grazing Areas

Pb pollution from industrial operations significantly impacts animal grazing sites, threatening both animal and human health. Industrial activities such as mining, smelting, battery manufacturing, and paint production release substantial amounts of Pb into the environment, affecting nearby ecosystems and grazing lands [58,60]. These contaminated grazing areas expose animals to Pb through ingestion of polluted vegetation, water, and soil, which poses a risk to human health through the consumption of animal-derived products.

One of the most significant contributors to Pb pollution is the mining and smelting of Pb-containing ores. Mines also contribute significantly to Pb contamination, as Pb often leaches into surrounding groundwater during mining operations. Mining processes release Pb into the environment through waste rock, tailings, and smelter emissions [61,62]. Pb is released into the atmosphere by smelting operations, then it gets deposited on surrounding soil, which contaminates grazing lands [24,45]. Another major source of environmental lead pollution is lead–acid battery manufacturing plants. Pb is released from battery manufacturing plants during the production process; the airborne Pb particles can travel a long distance, contaminating water resources and soil in nearby grazing areas [22].

Pb is also used in paint factories in pigment and drying agents, and these paint factories act as sources of Pb in the form of dust and pigments. The dust and particles settle on the soil, and animals grazing on Pb-contaminated grass take up this Pb, thereby increasing bioaccumulation of Pb in the food chain [1,5,61].

Pb contamination is also caused by waste incineration. Plastics, batteries, and electronic waste are all sources of Pb when incinerated; they release it into the atmosphere as either particulate matter or fumes, which eventually settle on the ground. This deposition results in the contamination of grazing lands, posing a risk to livestock that might later consume this Pb through grass without knowing that it is toxic material. Industrial recycling activities also contribute to Pb contamination, with a notable example being the informal recycling of electronic waste. Pb leaches from discarded materials into soil and groundwater, which animals use for feeding and drinking [47]. Smelting also contributes significantly to Pb contamination in grazing areas. The cumulative effects of industrial emissions from smelting, paint production, battery manufacturing, and waste incineration exacerbate the persistence of Pb in grazing areas due to its ability to bioaccumulate in ecological systems [47,63].

Animals that have been regularly exposed to high levels of Pb are at risk of serious health issues, such as neurological damage, stunted growth, kidney failure, and sometimes even death [64,65]. The ingestion of such animal products, particularly in children and pregnant women, is of great concern, as such factors increase health risks among the population [66,67]. In humans, exposure to Pb is associated with various negative health issues like cognitive decline, cardiovascular health issues, kidney troubles, and hypertension [64,68].

#### Contamination Routes from Industrial Sites to Grazing Areas

The pathways which cause the transfer of Pb contamination from industrial sites to grazing sites include airborne emissions, waterborne transport, and soil dispersal. The mechanisms listed below highlight how adjacent operations can transfer pollutants to nearby ecosystems, affecting animal grazing and human food sources.

Airborne emissions are a major contamination route. Particulate matter and volatile organic compounds released from industries are transported by wind and deposited on vegetation and soil in gazing areas [69]. Other means of Pb contamination include airborne pollutants dissolved in atmospheric moisture, which result in the formation of acid rain, which further spreads contamination [70,71]. Thus, animals grazing in Pb-contaminated areas are exposed to pollutants.

Water discharged from the Pb processing industry may enter into groundwater systems supplying agricultural land, contaminating plants and water sources essential for grazing animals [72,73,74]. Rainwater runoff from industrial sites may carry lead-contaminated soil particles to grazing areas, while sometimes soil erosion by wind or heavy rain may further pollute grazing land [75,76]. Thus, animals grazing on these lands may directly ingest contaminated soil, leading to bioaccumulation of Pb in their tissues [77,78].

Human actions further aggravate the spread of Pb contamination. Improper storage of hazardous materials, poor waste disposal, and inadequate transportation management can also contaminate grazing areas beyond safe limits [79,80,81]. People mostly dispose of dangerous materials in open spaces which are close to grazing areas. Table 4 shows short descriptions of contamination routes from industrial sites to grazing areas.

### 2.3. Deteriorating Infrastructure and Lead Pollution in Grazing Areas

A major aspect which causes Pb pollution in grazing environments is the deterioration of aging infrastructures, predominantly plumbing systems and water pipes made of Pb or containing Pb-based materials. Corroding structures may release Pb particles into groundwater and surface water, which contaminates soil and vegetation in nearby grazing lands [82]. A familiar example is the Flint, Michigan, water crisis, where the absence of corrosion control in Pb pipes triggered elevated Pb levels in the water supply, resulting in extensive public health issues [83].

In addition, industrial infrastructures during processing and manufacturing release Pb from products like batteries or paints, which can degrade over time. Thus, during gradual breakdown, Pb is released into surrounding environments, contaminating soil and water sources that grazing animals rely on [84]. Furthermore, poorly kept urban infrastructures, such as putrefying highways and deserted industrial areas, also contribute indirectly to Pb pollution from unregulated waste disposal, which increases Pb-based soil contamination in grazing lands [85].

### 2.4. Lead-Based Paint and Its Role in Roadside Contamination

Pb-based paint, once frequently used for its robustness, is a chief source of environmental contamination as it depreciates. The cracking and withering of paint on roadside structures releases lead into the soil and atmosphere, creating health risks for both humans and animals. Studies have shown a direct link between the close proximity of roadways with Pb-painted structures and increased Pb concentrations in nearby soil [86,87]. Contact with Pb-contaminated air and soil near these structures has extremely serious health consequences. For example, children living in zones with high traffic and worsening infrastructure exhibit higher blood Pb levels compared to those living further away from such environments [88,89]. It has also been reported that contamination caused by deteriorating Pb-based paint disturbs nearby ecosystems, as Pb leaches into water, which is detrimental to aquatic life and soil organisms [90,91]. Thus, the mechanism of Pb release into grazing lands and contamination of grazing areas operates through numerous pathways, hence causing risks to both animals and humans via the food chain [92].

## 3. Lead Exposure in Grazing Lambs

Researchers and livestock producers are very concerned about Pb exposure in grazing lambs because of the possible hazards to animal welfare and human health via the food chain [93]. Many management approaches, such as rotating grazing, obtaining uncontaminated feed, and adding minerals that prevent lead absorption to diets, have been put forth to reduce Pb exposure.

### 3.1. Pollution of Soil and Vegetation

A significant concern for grazing lambs is the build-up of Pb in roadside vegetation and soils, which directly affects their health [14]. The main source of Pb exposure is contaminated soil, as plants can absorb Pb from these soils, which can then be consumed by grazing animals [94]. Roadside soil Pb accumulation is mostly caused by human activity, including industrial and vehicle emissions and atmospheric deposition [95]. When car exhaust particles land on soil surfaces and link with organic matter or clay, the concentration of Pb in topsoil where grazing takes place rises [48]. Pb dust and lead-based paints are released into the environment due to the degradation of buildings and contaminate soil during heavy rains [1,96].

High levels of Pb can affect vegetation near roads in particular. Pb can be absorbed directly from the air or through polluted soil that plant roots can absorb [97]. The intake of certain plant species by lambs poses a significant health risk because of the physiological characteristics that make lambs more efficient at absorbing Pb. This has an impact on the animals and eating the meat from these lambs could be dangerous for people as well. Furthermore, many studies have reported that phosphorus- and calcium-rich soil improves Pb solubility, thus enhancing uptake by plants [98]. Therefore, it is essential to identify and resolve the causes of Pb contamination in grazing areas in order to safeguard food safety and preserve animal health.

#### Factors Affecting the Way Grazing Lambs Absorb Lead

One of the main factors influencing Pb accumulation in grazing lambs is soil contamination. The effect of tainted fodder is highlighted by the clear relationship found between lambs’ Pb levels and soil Pb concentrations [14]. Even seasonal fluctuations also matter in cases of contamination. During wetter seasons, lead bioavailability is greater, hence facilitating greater plant uptake [99]. Another important consideration is the age of lambs; due to their maturing gastrointestinal tracts and higher milk consumption levels, younger animals absorb lead more easily than adults [92]. Furthermore, ruminant dietary deficits in calcium and phosphorus might increase absorption of Pb, highlighting the significance of a balanced diet in reducing Pb uptake [16].

### 3.2. Ingestion Pathways: Oral Intake of Lead-Contaminated Soil and Vegetation

The primary ingestion pathways of sheep involve consuming plants and dirt from their surroundings, which can result in the bioaccumulation of hazardous substances like Pb. Sheep frequently engage in geophagia, or the deliberate ingestion of soil particles, which can make up between 10 and 30 percent of their daily diet of dry matter. They also graze close to the ground, increasing their interaction with soil [100,101]. As a result, any build-up of Pb in soil presents a serious concern because of this dietary pathway. Elevated Pb levels can be experienced by sheep that graze close to hazardous areas or eat feed cultivated in contaminated soils [102]. Because of atmospheric deposition and absorption through plant surfaces, foliage tends to collect larger quantities of Pb than roots, which makes it a particularly problematic environmental factor [97]. The extent of Pb accumulation in plant tissues is influenced by various factors, including plant species, age, growth stage, and environmental conditions [82].

### 3.3. Roadside Dust Contribution to Lead Exposure

Pb exposure in grazing sheep is largely caused by roadside dust. This dust contributes to environmental contamination since it contains Pb and other heavy metals [59,103]. Pb exposure is elevated in rural regions where sheep graze near roadways due to the frequent intake of contaminated dust and dirt [94]. This mode of administration has a significant impact on the bioavailability of Pb in sheep.

In addition to tire wear and vehicle exhaust, mining operations, trash disposal, and industrial production all contribute to contaminated roadside dust [45,58,61]. Pb can enter the bloodstream after consumption, attach to red blood cells, and reduce oxygen flow, which may result in anemia. Additionally, Pb particles can build up in the liver, kidneys, and bone, among other tissues, which can have harmful consequences on the health and reproductive capabilities of animals [10]. The possibility of contaminated dust and soil being consumed by sheep, which can result in a variety of health problems, is increased when grazing areas are close to busy roadways. Additionally, there is a significant correlation between the presence of heavy metals in grazing ground and being close to a road, which results in higher levels of Pb in grazing animals [9,84,104]. When contaminated meat and milk from affected animals are consumed, there may be health concerns for humans [105].

## 4. Pathways of Human Exposure

### 4.1. Pathway of Lead Bioaccumulation: From Contaminated Areas to Humans

A ubiquitous environmental pollutant, Pb can enter the human food chain via a number of different routes and cause bioaccumulation in both humans and animals. Soil contamination is a major pathway for Pb exposure, as it can accumulate in the environment due to both anthropogenic and natural geological sources [106]. Agricultural areas may be affected by this contamination, especially in areas where Pb concentrations are high, including mining sites or areas affected by industrial emissions.

More importantly, water is the main way through which Pb enters the food chain, Pb contamination in water is a serious environmental concern. Aquatic organisms that suffer from Pb-contaminated water are then eaten by higher trophic levels, such as humans and animals, resulting in bioaccumulation and biomagnification. Particularly among susceptible groups, this presents serious health hazards, including neurological and developmental problems. To protect ecosystems and public health, Pb levels in water must be monitored and controlled.

### 4.2. Soil to Plant Uptake

Crops in agricultural soils can absorb Pb, particularly root crops and leafy vegetables that have a high propensity to collect heavy metals. Plant roots mostly absorb Pb, and its distribution in plant tissues proceeds as follows: root > leaf > stem > grain [107]. This suggests that vegetables cultivated in polluted soil, especially those with deep root systems like potatoes, carrots, and parsnips, may acquire large concentrations of Pb that could be harmful to human health if ingested. Such crops are routinely grown in arable regions like those found in the Republic of Ireland, which include counties like Carlow, Wexford, and Dublin [108]. There have been reports of high Pb concentrations in the soil in several places there, particularly along the east coast [109].

### 4.3. Plant to Animal Transfer

Pb also bioaccumulates in livestock that graze on contaminated soils and plants. Ingesting contaminated feed leads to Pb absorption into the bloodstream of animals such as lambs. Pb, once ingested, binds with proteins and accumulates in various tissues, particularly in the bones, liver, and kidneys of animals [110]. Lambs, being herbivores, are at high risk of bioaccumulating Pb when grazing on contaminated land or ingesting contaminated water. This process leads to the eventual accumulation of Pb in the edible tissues of lambs, including muscle and organs, which are consumed by humans.

### 4.4. Animal to Human Transfer

When humans consume contaminated animal products, including meat or dairy, they are exposed to Pb. In humans, lead absorption mostly takes place in the gastrointestinal system, where Pb contends for absorption with vital minerals such as calcium and iron [111]. Because of its ability to imitate molecules, Pb can integrate into biological systems. For example, it can attach to proteins’ sulfhydryl (-SH) groups, which can alter enzyme activity, affect cellular metabolism, and increase oxidative stress [112]. Even at low concentrations, long-term exposure to lead can build up in soft tissues and bones, therefore causing systemic consequences including nephrotoxicity, neurotoxicity, and decreased hematopoiesis. Furthermore, because of their growing systems’ susceptibility and higher rates of absorption, some populations, like children and pregnant women in particular, are more vulnerable to the harmful effects of Pb. Exposure to lead at important developmental stages can result in permanent harm to the neurological system, which can manifest as delayed growth, behavioral problems, and cognitive deficiencies [18].

## 5. Impact of Lead on Lamb Health and Productivity

### 5.1. Lead Accumulation and Health Risks

Researchers have determined thresholds that are critical for the negative effects of Pb exposure on a lamb’s health. Only one liver sample above the 3 mg/kg criterion was found in a trial with lambs grazing close to a contaminated shooting range, indicating no immediate health hazards [113]. Conversely, studies conducted in mining areas found that 73.3% of sheep had blood Pb levels higher than the background norm of 6 µg/dl and that, in 68% of cases, concentrations in the liver exceeded the dangerous threshold of 5 µg/g [90].

### 5.2. Bioaccumulation and Productivity

Elevated tissue levels, especially in the kidneys, have been linked to chronic exposure to Pb pollution from mining, suggesting that the health effects may have an adverse influence on production [114]. Even though most muscle samples were below the allowable limit of 1 mg/kg, significant hematological abnormalities were found in a study on Lohi sheep, demonstrating that even low levels of Pb can have a negative impact on general health [114]. Thus, chronic exposure to excessive levels of Pb can result in serious health consequences, even though other evidence indicates that exposure to low concentrations of Pb may not pose immediate dangers. This highlights the need for continuous monitoring and risk assessment in contaminated environments.

### 5.3. Lead Toxicity and Physiological Effects in Lambs

Lead poisoning can have a serious negative impact on the physiology of livestock, especially grazing sheep. Usually, eating Pb-contaminated grass or soil or drinking contaminated water causes Pb poisoning [115]. According to Assi et al., prolonged exposure to Pb can cause serious health problems, such as stunted growth, impaired reproduction, neurological abnormalities, and even death [64]. Sheep are particularly susceptible to Pb poisoning due to their propensity to eat soil while grazing, particularly in areas with a history of industrial pollution or close to urban areas where environmental contamination from Pb-based products, like paint or gasoline, may occur [1,35,96]. Because of their vulnerability to dietary modifications and electrolyte fluctuations, sheep are especially vulnerable to increased absorption of Pb through the gastrointestinal tract [16].

After consumption, Pb builds up in the lungs, liver, kidneys, and bones, among other organs [116]. By blocking vital enzymes, it interferes with the heme production pathway, resulting in reduced oxygen carrying capacity, distorted red blood cells, and ultimately anemia [117]. Pb has the ability to pass across the blood–brain barrier, build up in the central nervous system, and interfere with oligodendrocyte and astrocyte growth and function. Due to oxidative stress, inflammation, and demyelination brought on by this impairment, nerve conduction is impaired [118,119].

Pb affects the function of the intestinal mucosa of the gastrointestinal system, deactivating digestive enzymes and causing malabsorption, diarrhea, and inflammation [120]. Furthermore, alteration of the gut microbiota may have long-term effects on immunological response and general health [121].

After Pb intake, it mostly attaches to red blood cells after it enters the bloodstream and builds up in soft tissues like the liver and kidneys. Higher content of Pb found in bones means past long exposure [122]. Research has indicated noteworthy altered organ function and elevated markers of oxidative stress after exposure to Pb, including lipid peroxidation and reactive oxygen species (ROS) [123]. Additionally, increased infant death rates, reduced fecundity, and reproductive problems have been seen in sheep exposed to Pb [64,124,125,126].

### 5.4. Impact on the Central Nervous, Hematological, and Immune Systems

The central neurological, hematological, and immunological systems of lambs are greatly impacted by Pb intoxication [127]. The developmental neurotoxicity that resembles symptoms seen in children exposed to Pb in humans is one important cause for concern [127]. Reactive oxygen species (ROS) produced by lead-induced oxidative stress damage cells and cause apoptosis, which impairs brain growth and function [128]. Some of this damage may be mitigated by antioxidants such as vitamin E [129]. Pb interferes with essential cellular processes in the central nervous system, such as ion transport and neurotransmitter production [130]. Pb poisoning can cause headaches, convulsions, cognitive decline and, in extreme situations, even death [125].

The hematological effects of Pb toxicity are mainly caused by its inhibition of important enzymes that interfere with heme synthesis, which results in anemia marked by an increase in immature red blood cells and hemolysis [127,131,132]. Lead has a negative impact on the immune system as well, causing oxidative stress and altering signaling pathways like the nuclear factor (NF)-κB pathway that are essential for immunological function [133]. Reduced immunoglobulin synthesis and impaired immunological responses are the outcomes of this impairment [134].

### 5.5. Growth and Developmental Implications

Studies show that exposure to Pb might have a negative impact on the growth and reproductive health of lambs. Elevated blood Pb levels can impede growth by causing anemia and obstructing the transport of oxygen to tissues [90,134,135]. Furthermore, exposure to Pb causes DNA damage and oxidative stress, which further impede cellular growth and activities [136]. Pb poisoning also impairs reproductive function by upsetting hormone balance and endocrine function, which leads to irregular estrous cycles, lower fertility, and a greater rate of abortions [124,126]. Due to placental transfer, pregnant ewes exposed to Pb may give birth to lambs that are smaller than usual or develop congenital abnormalities [116,137].

Disruptions in cognitive function and social development are among the long-term effects on lamb productivity, especially if exposure happens during pregnancy or the early stages of life [138,139]. Furthermore, exposure to Pb has an impact on the reproductive systems of both males and females, resulting in decreased lambing percentages and worse quality sperm [140].

### 5.6. Transfer to Lamb Products and Consumer Health Risks

Lead bioaccumulation in lamb tissues puts consumers’ health in serious danger. Contaminated meat can introduce Pb into the food chain and eating it can result in both immediate and long-term health problems such anemia, renal failure, and neurological diseases [10,141]. According to Al Osman et al. and Sahu et al. children who eat lamb products contaminated with lead run a higher risk of neurological diseases, which can hinder cognitive development and result in learning difficulties [142,143]. Adults who are exposed to long-term high levels of Pb may experience anxiety, memory loss, and higher chances of developing long-term illnesses including Parkinson’s and Alzheimer’s diseases [144,145]. Additionally, Pb impairs renal function and may cause renal disorders and lower glomerular filtration rates [146]. Pb-contaminated lamb products can have a negative impact on fetal development in pregnant women, which may lead to low birth weights and long-term cognitive problems in the children [137,147].

### 5.7. Lead Levels in Blood and Liver: Potential Health Effects for Sheep

Animal cases of Pb poisoning are common, especially for animals that graze in contaminated areas [148,149]. Pb levels in the blood and liver can track Pb build up and health impacts and they are trustworthy markers of environmental exposure [150]. According to Tesi et al., the geometric mean of Pb levels in ewes from a mining area was 6.7 μg/dL, indicating subclinical Pb poisoning. In contrast, blood Pb levels in reference animals in the investigation were within the background range for cattle (≤6 μg/dL) [12,151].

Blood Pb levels consistent with subclinical poisoning were found in 77.3% of sheep from the mining area (100% of rams and 63% of ewes). The sampling location has a history of active mining, likely exposing the sheep to elevated Pb levels in soil, plants, and water [152]. Seasonal variations may explain the higher prevalence of subclinical exposure observed in this study compared to previous reports from the same area. When sampling was performed during summertime, a period when sheep may ingest more soil due to limited pasture availability, mean blood Pb levels of 5.15 μg/dL were found in sheep exposed to Pb-polluted feed [153], while another researcher found a median of 14.7 μg/dL in sheep grazing in contaminated pastures [154]. A negative correlation between blood Pb levels and age was noted, suggesting that younger animals may be at higher risk due to increased gastrointestinal absorption or bone turnover. Liver Pb concentrations in sheep sampled from the reference site showed Pb concentrations of ≤0.5 μg/g d.w, while liver lead concentrations were found of 6.16 μg/g d.w in sheep from a mining area, which is 29 percent higher than the reference site. An alarming 93.8% of sheep from the mining site had liver Pb levels exceeding this baseline level, with 68.8% surpassing the minimum toxic threshold (5 μg/g d.w.). Phillips et al. reported that chronic Pb exposure in livestock could lead to alterations in physiological systems. The mechanisms of Pb poisoning include enzyme inhibition, oxidative stress, and interference with heme synthesis. Prenatal Pb exposure at low levels (10–15 μg/dL) is linked to significant developmental issues [153]. Clinical symptoms, such as anemia and weakness, may manifest at blood Pb levels similar to those found in this study [155].

### 5.8. Lead Levels in Liver and Muscle: Implications for Public Health

Subclinical chronic Pb exposure in livestock can result in residual levels in animal products that exceed legal limits, posing risks to human health [156]. The EU has established Maximum Residue Limits (MRLs) for livestock: 0.5 μg/g w.w. for offal (1.4 μg/g d.w.) and 0.1 μg/g w.w. for meat (0.34 μg/g d.w.) (EC 629/2008) as shown in Table 5. Results have shown that 87.5% of sheep from a mining area had liver Pb levels exceeding the EU MRL for offal, while muscle samples did not exceed the MRL for meat. However, muscle Pb levels were double those of a reference farm, with one animal approaching the EU MRL.

## 6. Mitigation Strategies

Lead contamination in roadside grazing lambs poses serious health risks, making the implementation of effective mitigation strategies essential. Various research findings suggest several approaches that can significantly reduce lead exposure and its adverse effects on lamb health.

### 6.1. Chelation Therapy

Chelation therapy is one of the most effective ways to treat livestock lead toxicity. Chelation medications are given as part of this treatment to bind to lead and encourage its elimination from the body [127]. Furthermore, it has been shown that mineral supplementation, especially using commercial mineral blocks, is effective in reducing sheep blood Pb levels. By increasing Pb excretion in the feces, this technique lowers exposure levels overall [16]. In order to mitigate Pb exposure in lambs raised on roadsides, certain fence and grazing management techniques are necessary. According to research, deliberate actions can considerably reduce the amount of Pb that animals are exposed to in contaminated surroundings.

### 6.2. Use of Mineral Blocks (MBs)

It has been demonstrated that supplementing grazing lambs with mineral blocks high in calcium and phosphorus can reduce Pb bioavailability by up to 88% in vitro [16]. In practical applications, MBs significantly lowered blood Pb levels in lambs, demonstrating their effectiveness in contaminated environments.

### 6.3. Soil Ingestion Management

Although MBs aid in reducing Pb absorption, they do not lessen soil ingestion, which continues to be a crucial pathway for Pb exposure [16]. For comprehensive management, it is therefore recommended to combine MB supplementation with fencing strategies.

### 6.4. Soil Remediation: Techniques for Reducing Lead Levels in Contaminated Soil

Varieties of strategies to successfully reduce Pb concentrations are required for the management and cleanup of Pb contaminated soils. Chelation is used to remove heavy metals from soil by using chemical solutions based on water [157]. Usually, this procedure includes chelating agents like citric acid, ethylenediaminetetraacetic acid (EDTA), or organic acids. These substances combine in the soil, enabling solid particles to be separated from Pb-containing fluid, hence resulting in a soil matrix that is less polluted [158]. Although soil cleaning has been demonstrated to successfully lower Pb levels, it may unintentionally eliminate vital nutrients and trace elements from the soil [157,158].

Phytoremediation, which uses plants’ innate ability to absorb, collect, or change pollutants, is another successful remediation technique. Some plant species, such as Indian mustard (*Brassica juncea*), sunflowers (*Helianthus annuus*), and reed canary grass (*Phalaris arundinacea*) have demonstrated significant potential for removing lead from contaminated soils [159]. Over time, the phytoextraction process can progressively lower the amount of Pb in soil. However, the drawbacks include its sluggish metal removal rates and possible hazards when disposing of contaminated plant materials [160].

Another approach that shows promise for treating Pb contaminated soils is electrokinetic remediation. This method uses a direct current electric field to move pollutants through soil pores and to specified collection locations [161]. Electrolyte buffering or the addition of chelating agents can improve the process efficiency. Although electrokinetic remediation has demonstrated promising results in lab environments and pilot programs, soil heterogeneity and the requirement for careful process optimization may present difficulties [162].

Geomicrobiological treatment is a unique approach to soil remediation that involves interactions between the physical and biological components of the system [163,164]. This biological approach makes it possible to treat contaminated soils in situ effectively when paired with mineral supplements. To improve the effectiveness and usefulness of geomicrobiological remediation, more research is necessary.

### 6.5. Fencing and Grazing Management

In order to protect the health of lambs and ensure the safety of the meat they produce, fencing is an essential management strategy that keeps them out of contaminated areas [165,166]. Fencing is a critical component in lowering the amount of hazardous components lambs are exposed to by keeping them out of Pb-contaminated areas. Farmers can successfully manage animal movement and grazing patterns, while avoiding disturbances to natural behaviors, by strategically implementing fencing. Farmers can greatly reduce needless exposure risks by establishing fenced-off areas around known or suspected Pb-contaminated locations and routinely checking soil and plant lead levels. Boundary-line grazing systems are an additional option that can be used in conjunction with this. They use hedges or other extra obstacles to help with regulated grazing. This technique lowers the hazards for lambs kept in specific pasture patches and reduces the spread of parasites. Fencing can be implemented, but there are drawbacks, such as the cost of upkeep and installation, especially for large-scale farms that deal with high levels of pollution. Furthermore, worries about animal welfare could surface, which would call for more investigation into different approaches to controlling Pb exposure in lambs.

### 6.6. Rotation of Grazing Areas and Alternative Foraging Options

A good management technique to reduce exposure to Pb-contaminated soils is rotational grazing. Sheep are routinely moved between pastures in order to promote fodder regrowth and reduce the dangers of overgrazing and environmental contamination. Sheep can spend less time in any one place by moving between smaller paddocks, which reduces their exposure to Pb [167]. Rotational grazing, which usually includes splitting a pasture into smaller paddocks for movement every few days, is dependent on a number of parameters, including pasture area, carrying capacity, and meteorological conditions [168].

Additionally, a long-term plan for recovering contaminated pastureland can involve phytoremediation. Using particular plant species that are well known for their capacity to either accumulate Pb in their tissues or improve metal immobilization in the soil, these processes take advantage of the Pb-absorbing properties of soil [169].

Furthermore, adding mineral supplements to the diets of sheep could reduce the amount of Pb that enters their digestive processes. Pb absorption can be reduced by minerals that compete with Pb for gastrointestinal tract absorption sites, such as calcium and iron [16].

For rotational grazing management to be effective, it is important to monitor the amounts of pollutants in soil and vegetation. Frequent soil and forage testing assist in identifying high-risk regions where exposure needs to be reduced. This information enables farmers to choose suitable substitute foraging areas and create well-organized rotational grazing plans that successfully reduce the hazards associated with Pb exposure.

### 6.7. Challenges and Effectiveness of Soil Remediation

Soil remediation is essential for dealing with contamination. Various options which can be employed for soil remediation include stabilization/encapsulation, phytoremediation, and excavation and disposal [170]. A number of criteria, including cost-effectiveness, site features, and environmental considerations, play a role in the method selection process. For example, due to much higher expenses, excavation may not be possible in large, contaminated areas, such as agricultural fields.

Accurately determining the level of Pb pollution at grazing locations is another difficulty. Depending on the kind of soil and the build-up of pollutants over time, Pb distribution can change within a location [46]. An extensive sampling plan is essential for doing an accurate impact assessment. Moreover, Pb mobility and bioavailability are influenced by soil characteristics such as texture, pH, and organic matter concentration [127,171]. Due to their intricacy, these features must be carefully considered in the remediation planning stage.

Despite these obstacles, Pb-contaminated soil in sheep grazing areas can be remedied using practical methods. One technique that effectively lowers Pb bioavailability is stabilization/encapsulation, which changes the metal into less soluble forms [169]. Applying amendments, such as phosphate-rich materials or biochar, can help to reduce Pb soil contamination [172,173]. Pb ions are less likely to be released or absorbed by plants after binding with these substances. Another method that is becoming more and more popular for remediating soil is phytoremediation, especially in agricultural settings. In addition to its low environmental impact and cost-effectiveness, this approach provides benefits including soil stabilization and the restoration of ecosystem services [169,174].

## 7. Monitoring and Risk Assessment

### 7.1. Development of Monitoring Programs to Assess Lead Contamination

To assess Pb pollution in the soils of sheep grazing locations, monitoring plans and programs must be established. The main objectives of these projects are to evaluate Pb concentrations, locate sources of contamination, and reduce the negative health impacts that eating sheep products can have on both humans and sheep. A crucial stage in developing a monitoring program is deciding on suitable sampling methods. Because composite soil sampling provides a representative overview of contamination levels across a greater area, it is frequently used [175]. In order to account for vertical Pb distribution brought about by environmental processes such as soil mixing and erosion, samples should be obtained at different depths [176].

Pb concentrations in soil samples are mostly determined by laboratory analysis. Inductively Coupled Plasma Mass Spectrometry (ICP-MS) is a widely used technology because of its great sensitivity, accuracy, and precision [177]. Furthermore, using geochemical indices can make it easier to track temporal and spatial patterns in lead contamination at various grazing sites [178]. In addition to soil sampling, animal tissue analysis must also be monitored. Without this investigation, it is difficult to diagnose subclinical Pb toxicity in cattle. Scientists can determine the Pb concentrations in afflicted animals by evaluating liver and kidney samples [179]. Sheep blood samples can offer important information about recent exposure to Pb and its possible health effects. The monitoring process can be greatly improved by the inclusion of latest Geographic Information System (GIS) softwares, such as ArcGIS Pro (Version 3.4). Prioritizing intervention efforts in impacted grazing areas is made possible by mapping the spatial distribution of Pb-contaminated soils [180]. Time-series mapping can also assess the efficacy of remediation measures that have been put in place and uncover seasonal patterns. Throughout a monitoring program’s inception, communication with pertinent stakeholders, such as government agencies, researchers, farmers, and veterinarians is very important. This kind of cooperation encourages the development of best practice and policies meant to protect sheep populations from Pb contamination. Attaining the intended results of any monitoring program also depends on effective communication.

### 7.2. Risk Assessment for Grazing Lambs and Consumers

In order to evaluate Pb pollution in sheep grazing places and ensure the health and safety of both humans and livestock, risk assessment techniques are important. A number of techniques can be used, such as soil testing, bioavailability assessment, toxicity reference values, and Geographic Information Systems (GIS) mapping.

The first step in identifying Pb pollution in grazing areas is soil testing [171]. Scientists can learn more about the distribution and concentration of Pb on a site by gathering representative soil samples. The possible health concerns can then be assessed by comparing the results to established criteria, such as those issued by the US Environmental Protection Agency (EPA). While Pb concentrations may be determined through soil testing, Pb bioavailability must also be considered when evaluating concerns. Bioavailability is the percentage of a pollutant that an organism may take up from its surroundings [181]. In order to assess bioavailability, plant samples from grazing sites must be gathered, especially those eaten by sheep, and their Pb uptake must be examined. Determining the amount of Pb taken up by these plants and then consumed by sheep is essential for evaluating possible health hazards and the financial effects on farmers. Reference levels for toxicity are still another essential element of risk assessment. The toxicity levels of several species have been established by the EPA, considering their vulnerability to various pollutants, including Pb. Sheep, for instance, show varying tolerances to Pb exposure in comparison to people or cattle [182,183]. It is feasible to quantify the prospective health hazards that sheep in contaminated grazing areas may encounter by comparing these toxicity reference levels with measured lead concentrations in soil and the findings of bioavailability studies.

Additionally, GIS mapping can be extremely important for risk assessment techniques. With the aid of this technology, scientists may evaluate potential exposure paths for animals at grazing areas, analyze the spatial distribution of pollutants, and pinpoint hotspots [180]. Health risk assessments are made more thorough by combining Pb concentration data with GIS mapping. This helps decision-makers manage cattle movement or put remediation plans into place to reduce exposure to polluted soils.

### 7.3. Regulations and Public Health Concerns

Soil contaminated with Pb poses a serious threat to human health, especially in places where sheep graze. Numerous restrictions have been put in place to combat the negative impacts of Pb on both the environment and human health [183]. The purpose of these rules is to protect the health of people who eat sheep products as well as sheep.

To control Pb concentrations in soil and grazing areas, numerous national and international rules have been implemented. A guideline value of 400 parts per million (ppm) has been established by the US Environmental Protection Agency (EPA) for Pb levels in soil in residential areas where children may be present. However, there are no particular rules for grazing areas or agricultural grounds. On the other hand, several nations have set legal restrictions for agricultural soils. For instance, the European Union has allowed Pb levels in agricultural soils to range between 50 and 300 mg/kg [184]. A summary of regulations and limits on Pb levels is provided in Table 6.

Maximum permitted limits for Pb in food and animal-derived products have been imposed in several countries to protect public health and guarantee food safety. For example, the European Commission set limits of 0.1 ppm for beef and 0.05 ppm for offal [184]. Furthermore, the maximum levels of Pb in milk and dairy products, which range from 0.01 to 0.02 ppm, have been defined by the Codex Alimentarius Commission. It is essential to conduct routine testing on soil and goods obtained from animals to guarantee adherence to these rules and safeguard public health. Remedial procedures can be used if Pb levels in soil surpass regulatory thresholds or if they pose a risk to grazing animals and their products.

These techniques could involve removing contaminated soil mechanically, adding chemical amendments (such as phosphates), or stabilizing the soil with different additives to immobilize Pb [185,186,187]. As was already mentioned, by preventing sheep from ingesting too much dirt, management techniques like switching up grazing sites and providing them with water and feed supplements can also help to decrease Pb exposure.

### 7.4. Regulatory Standards

Ensuring sustainability and safety in grazing situations requires adhering to regulatory criteria for Pb levels. These regulations are designed to reduce the danger of Pb exposure in cattle and reduce the amount of Pb that humans may consume. Different nations have different standards for soil quality, which reflect differences in goals and approaches. Acceptable Pb concentration levels are often set by environmental agencies using background data and risk evaluations that consider possible transfer pathways.

Pb regulations in sheep grazing areas are essential for protecting both public health and animal welfare. Although national frameworks may vary, they consistently aim to preserve the quality of soil and reduce the dangers of exposure for both people and animals. Around the world, better management practices and continuous monitoring and assessment are needed to address Pb contamination in grazing areas.

## 8. Comparison of Lead Contamination Limits in Livestock Products

The possible harmful consequences of Pb contamination on human health upon ingestion make it a major public health concern when it comes to cattle products. The need for agreement to improve public safety is shown by comparing the Pb contamination limits across different worldwide standards, which emphasizes the disparities in regulatory systems. For those who handle and process contaminated products, Pb pollution presents occupational dangers in addition to dietary risks. While exact recommendations frequently depend on evaluations by regional regulatory agencies, the World Health Organization (WHO) offers guidance for limiting Pb exposure among agricultural workers [188,189].

### Evaluating the Adequacy of Current Regulations for Consumer Health Protection

Although regulations pertaining to Pb contamination in livestock products have been put in place to protect consumer health, questions still exist regarding their effectiveness. This analysis aims to assess the degree to which current regulations protect consumers from Pb exposure from livestock products. Pb contamination is a serious concern for consumer health because excessive exposure to this highly toxic heavy metal can lead to serious health problems, such as neurological disorders and developmental issues, especially in young children. Therefore, strong regulations are essential for reducing the risk of Pb exposure through the consumption of livestock products, including among children and expectant mothers. Regulations that are in keeping with these principles can improve consumer protection. It is also essential that these regulations be implemented and upheld. Enough surveillance and monitoring systems need to be in place to guarantee adherence to set guidelines. To detect noncompliance and take appropriate action, it is imperative to conduct routine Pb contamination testing on cattle products. Furthermore, efficient traceability and regulatory supervision can be facilitated by animal farmers maintaining accurate records. Another important factor is consumer understanding of the dangers posed by Pb contamination in cattle products. Education programs and public awareness efforts can enable customers to choose these items with knowledge. This includes guidelines for safe handling and preparation, as well as information on possible sources of Pb contamination and unambiguous labeling.

Furthermore, cooperation between many stakeholders is essential for handling Pb contamination successfully. Collaboration between governmental organizations, livestock farmers, business associations, and academic institutions can promote a thorough grasp of the issue and result in the creation of practical mitigation plans. Although the present laws are designed to prevent cattle products from contaminating consumers with Pb, doubts remain over their effectiveness. It is imperative to guarantee that regulatory restrictions correspond with health-based guidelines and to carry out efficient implementation, monitoring, and public awareness campaigns. Moreover, promoting cooperation among interested parties can help create all-encompassing plans to reduce Pb exposure in cattle products.

## 9. Communication and Awareness

### 9.1. The Role of Public Awareness Campaigns in Educating Farmers and Consumers

By informing farmers and consumers about the risks involved and offering alternative solutions, public awareness programs play a critical role in reducing Pb emissions and contamination along highways [190]. By raising awareness of the causes, consequences, and pathways of Pb contamination, these programs enable people to make decisions that minimize environmental pollution.

These kinds of efforts help to create awareness among the public, which can lead to an understanding of tackling Pb poisoning. In the interest of both farmers and consumers, various platforms, including social media, print media, radio broadcasts, television commercials, seminars, and workshops can be employed. For example, many farmers might not be aware that using leaded gasoline in farming equipment adds to Pb pollution in the atmosphere, which then ends up on plants, soils, and water and nearby residential areas. Awareness campaigns on these issues can encourage farmers to adopt cleaner technologies, such as unleaded fuel or electric-powered machinery.

Additionally, these programs encourage cooperation between farmers and customers in using unleaded items in the pursuit of a common objective. Customers may encourage understanding and collaboration with farmers by providing them with information about the health risks associated with Pb exposure, including neurological impairments and cognitive difficulties. Customers might decide, for instance, to support nearby farmers who use organic fertilizers enhanced with vital micronutrients or crop rotation, two examples of environmentally beneficial farming methods. All parties involved benefit from the mutual support networks this collaboration builds, which promote healthier behaviors.

Campaigns for public awareness may also have an impact on governmental decisions. People can put pressure on legislators to pass suitable laws and regulations targeted at lowering Pb emissions and pollution as they become more aware of the threats that Pb exposure poses to human health and the environment. For example, greater public knowledge could result in the implementation of recycling programs, rules governing agricultural operations, or more stringent car emission regulations.

Lastly, these efforts support continued investigation and creativity in the field of Pb reduction. They inspire academics and politicians to create innovative technologies, remediation methods, and pollution monitoring plans by drawing attention to the issues around Pb contamination. An example of how increased awareness might result in workable solutions against this dangerous metal is the use of biochar soil additives by farmers to reduce Pb toxicity in crop production [191].

### 9.2. Initiatives to Reduce Lead Emissions and Contamination Along Highways

Due to the detrimental effects of Pb exposure on human health, particularly in children, Pb emissions and contamination along highways represent severe public health hazards [192]. Below are a number of practical steps that can be taken to address this problem and lower Pb pollution and emissions.

Phasing out leaded gasoline: In spite of global initiatives to eliminate leaded gasoline, usage of the fuel still exists in some areas, mainly the Middle East [193]. Leaded gasoline can be phased out to greatly lower airborne Pb particle pollution from automobiles. Legislators ought to support measures that encourage the use of only unleaded gasoline.

Green barriers: Planting hedges or vertical gardens beside roadways can aid in the absorption of Pb particles and other air pollutants. For example, ferns, which are epiphytes, can absorb nutrients and pollutants directly from the atmosphere, acting as potential mitigators of pollutants. Their fronds trap particle-bound heavy metals, making them ideal candidates for biomonitoring and improving air quality. *Nephrolepis exaltata* (Boston Fern): known for its high capacity to capture air pollutants, including heavy metals. *Pteris vittata* (Chinese Brake Fern): recognized for its phytoremediation abilities, especially for arsenic, is also effective for Pb [194]. Urbans trees like poplars, willows, and oaks are effective at filtering airborne heavy metals due to their dense canopies and high transpiration rates [195]. These barriers improve air quality and enhance the aesthetic appeal of the environment.

Frequent road dust cleaning: Using vacuum sweeping trucks, roads and highways can be kept cleaner and less susceptible to Pb-containing dust. A methodical approach to road upkeep is essential for lowering airborne levels of hazardous metals close to roadways.

Putting traffic management policies into practice: Carpool lanes, congestion pricing, and enhanced public transit are some of the strategies that can assist in lowering the number of vehicles on the road close to highways, which will minimize the number of dangerous emissions, such as Pb pollution.

Using water controls: Since water serves as the main means of transportation of lead and other heavy metals through ecosystems, water quality controls in grazing areas near to highways are very important for reducing Pb transmission. Heavy metals can bioaccumulate from contaminated irrigation or livestock water, affecting animal health and potentially finding their way into the human food chain. Pb runoff can be considerably decreased and grazing areas near highways can be protected with regular water quality controls. This procedure is essential for maintaining consumer safety and the environment.

Public awareness campaigns: It is important to inform communities next to highways about the dangers of Pb exposure and to offer practical advice on how to reduce those hazards. Mass media campaigns, workshops, and community outreach initiatives are among the tools public health officials can use to increase awareness and educate locals about steps they can take to minimize exposure, such as using correct cleaning methods and keeping an eye on soil conditions in their backyards.

## 10. Prospects for Future Research Paths

The problem of Pb pollution in grazing habitats requires a number of research and intervention approaches, including improved data collection, monitoring of Pb exposure mitigation approaches, comprehending the biological pathways of Pb poisoning, management techniques for grazing, and implications for public health and policy. We should plan upcoming studies which develop real-time soil and air quality sensors as well as monitoring systems to estimate Pb levels in grazing regions. The introduction of these technologies can help us to identify Pb hotspots, thus early action can be taken to safeguard cattle. Additional research into the molecular pathways that lead to interference with lamb’s physiological systems may reveal biomarkers for early detection of Pb poisoning.

The long-term advantages of grazing practices including rotational grazing, pasture resting, and boundary-line grazing in lowering animal Pb intake require more investigation. It could also be helpful to investigate cutting-edge methods like bioremediation using hyperaccumulator plants, which remove Pb from contaminated soil. Participatory research and teaching programs that involve farmers can improve grazing techniques and encourage the adoption of more sustainable land-use practices, particularly in areas with high traffic or industrial activity.

Stricter regulatory criteria for environmental Pb concentrations should be established, especially in agricultural areas. Maintaining a continuous communication channel among scientists, policymakers, and industrial stakeholders is crucial for tackling Pb poisoning at its origin. Public awareness efforts ought to highlight the dangers of Pb poisoning in food items and encourage the safe handling of livestock in polluted areas. To safeguard customers, food-labeling transparency may also be required, especially for livestock kept close to roadways.

## 11. Conclusions

Pb is a heavy metal responsible for various dangerous diseases; lambs exposed to Pb pollutants from grazing environments are under significant threat. Pb ingested from contaminated soil, water, and from foraging in areas near to highways causes toxic effects in lambs, therefore compromising their productivity, immune function, and overall well-being. If there is persistence of Pb exposure in lambs, it causes long-term risks to livestock. The mechanism of Pb toxicity is so complex that it affects most organs, causes many changes, such as impaired physiological functions, and causes oxidative stress, enzyme inhibition, and various interferences with calcium-dependent processes. Pb toxicity causes neurotoxicity, hematological impairments, and immunosuppression in lambs, thus causing decreased productivity, reproductive health issues, and even death in severe cases. Additionally, Pb is reported to reach the tissues of lambs exposed to contaminated grazing areas and is subject to bioaccumulation processes, which poses a direct threat to humans.

To mitigate the risks caused by Pb contamination, an interdisciplinary approach is required involving environmental science animal husbandry, toxicology, and public health experts. Due to the complex paths leading to Pb contamination, which include industrial processes, atmospheric deposition, human behavior, and vehicle emissions, complete remedies must incorporate ecological, governmental, and community-based tactics. Prospective investigations ought to assign precedence to comprehensive, multidisciplinary methodologies that consider the intricacies of Pb pollution in grazing habitats. We cannot guarantee the long-term sustainability and safety of animal production systems, protect human health, and maintain the integrity of ecosystems unless we integrate environmental science, toxicology, veterinary medicine, and public policy.

## Figures and Tables

**Table 2:** Major sources and details of lead contamination.

Source	Details	References
Leaded Gasoline	Widely used in the past, phased out due to health concerns, major source of soil pollution.	[37]
Diesel Exhaust	Incomplete fuel combustion and oil additives release lead; regulations have reduced emissions, but traces remain.	[41]
Mining and Smelting	Waste rock, tailings, and smelter emissions contribute significantly to soil contamination.	[45]
Lead–Acid Batteries	Lead particles released during production travel long distances before settling on soil and water bodies like lakes and ponds.	[22]
Paint Production	Lead-based pigments and drying agents released during production contaminate surrounding soil.	[46]
Waste Incineration	Lead in batteries and e-waste volatilizes during incineration and contaminates surrounding areas via atmospheric deposition.	[47]

**Table 3:** Factors influencing lead dispersion near highways.

Factor	Impact	References
Traffic Density	Higher traffic volume results in increased lead emissions and greater lead accumulation near roads.	[50]
Vehicle Type	Older vehicles release more lead, particularly those using leaded gasoline.	[19]
Emission Control Technology	Technologies like catalytic converters reduce emissions, but not completely.	[55]
Meteorological Conditions	Wind speed, direction, temperature, and humidity affect how far lead particles are carried and where they deposit.	[56]
Land Use Near Highways	Vegetation can filter out lead, while impervious surfaces cause Pb to wash off and contaminate rivers.	[57]

**Table 4:** Contamination routes from industrial sites to grazing areas.

Route	Description	References
Airborne Transport	Particles emitted by industrial processes and dispersed by wind, settling on soil, water, and vegetation.	[69]
Waterborne Transport	Contaminants discharged into water bodies like streams, rivers and lakes or leached into groundwater spread through the water cycle, affecting animal grazing sites.	[74]
Soil Transport	Polluted soil particles carried via runoff or erosion to nearby areas, including grazing land.	[78]
Human Activities	Improper waste disposal, transportation, and landfill operations contribute to lead pollution spreading into grazing areas.	[81]

**Table 5:** Lead levels in sheep from mining vs. reference sites.

Measurement	Mining Area (Mean)	Reference Site (Mean)	Percentage Exceeding MRL
Blood Pb (μg/dL)	6.7	≤6	77.3%
Liver Pb (μg/g d.w.)	6.16	≤0.5	93.8%
Muscle Pb (μg/g d.w.)	0.28	0.14	None exceeded MRL

**Table 6:** Summary of regulations and limits on Pb levels.

Category	Regulatory Body	Limit
Soil in Residential Areas	US Environmental Protection Agency	400 ppm
Agricultural Soils	European Union	50–300 mg/kg
Beef	European Commission	0.1 ppm
Offal	European Commission	0.05 ppm
Milk and Dairy Products	Codex Alimentarius Commission	0.01–0.02 ppm

## Data Availability

No new data were created.
